# A Method of Using Cocaine for Local Anæsthesia

**Published:** 1894-12

**Authors:** 


### A Method of Using Cocaine for Local Anæsthesia.

Krogius (*Centralbl f. Chir.*) describes a new method of producing cocaine analgesia, which is based on the fact that when a solution of this agent is injected into the subcutaneous tissue near to a nerve trunk it causes loss of sensation over a large zone corresponding to the peripheral distribution of this nerve. In order to reach the selected nerve trunk with certainty, and to apply the cocaine to several of its branches at the same time, the author, in injecting the subcutaneous tissue, passes his needle across the long axis of the limb, and as the needle is thrust along, the solution is gradually discharged. An injection made in this way across the root of a finger will, in the course of ten minutes, result in the analgesia of the whole digit, not of the skin only, but also of the tendons, the periosteum, and all the deep structures. If one or two injections be made transversely near the wrist, a considerable extent of the palm of the hand may be thus rendered analgesic. The sensibility of the ulnar side of the hand as far as the roots of the last two fingers, may, it is stated, be abolished by injecting a solution of cocaine over the ulnar nerve at the back of the elbow. By injecting over both supraorbital notches, analgesia may be produced in the whole of the middle portion of the forehead. The analgesia caused by this method of using cocaine attains its greatest intensity and extent for five to ten minutes after the injection, and is maintained for a quarter of an hour or even longer. The author injects only a weak (two per cent.) solution of cocaine, and keeps the patient recumbent for at least a quarter of an hour after the operation. This method has been practiced with success at Helsingors in 200 minor operations, such as amputation of the fingers and toes, excision of palmar fascia, and phimosis.—*British Med. Journal.*

